# Cause of Dental Caries

**Published:** 1878-02

**Authors:** 


### ARTICLE VI.

### *Cause of Dental Caries.*

Mr. A. Stewart, in the *British Medical Journal*, treats this subject very correctly, as follows:—

The general prevalence of dental caries is chiefly owing to food remaining on and between the teeth after meals—from breakfast time till the following morning—when, according to custom, the teeth are brushed; brushed, but probably not cleaned, as the brush is more often used to polish the surface merely than to assist in removing what has accumulated between them. Experiments have been referred to that prove the solvent action of weak acids on the teeth; and I think it will be conceded without proof that, were portions of our ordinary food, mixed and moistened as in mastication, kept during the night at the high temperature of the mouth, the compound would be sour. It follows that dental caries must continue to prevail as now, while it is the custom to allow the food to remain in contact with the teeth all night.

The following observations show the dependence of caries on food remaining in contact with the teeth. When the teeth are wide apart food is not retained, and they generally remain free from caries. The lower front teeth are seldom attacked by caries when, as is generally the case, the spaces between are closed to the entrance of food by tartar. The backs of all the teeth, upper and lower, being kept free from food by the tongue, are seldom affected by caries. Lodgment of food takes place between the bicuspids, between the molars, in the depressions on the masticating surface of these teeth, and on the buccal walls of the molars, and these are the chief seats of caries. While mastication is performed by the molars and bicuspids, the upper front teeth remain free from food and from caries; but, when they themselves are made to do the work of lost or diseased molars, and the food gets between them, caries is certain to follow before long. Further proof cannot be required that, if no food remained in contact with the teeth after eating, they would be free from caries, unless acted on by acidity from other sources. The only indications, therefore, for the prevention of dental caries are the neutralization of acid applied to the teeth and the removal of food before it has become acid. The food should be removed after every meal, and all who have not the opportunity of doing so should not fail to remove it thoroughly every night at bedtime by rinsing, as the brush cannot be trusted to remove the food from between the teeth.

